# Development and Assessment of an Interpretable Machine Learning Triage Tool for Estimating Mortality After Emergency Admissions

**DOI:** 10.1001/jamanetworkopen.2021.18467

**Published:** 2021-08-27

**Authors:** Feng Xie, Marcus Eng Hock Ong, Johannes Nathaniel Min Hui Liew, Kenneth Boon Kiat Tan, Andrew Fu Wah Ho, Gayathri Devi Nadarajan, Lian Leng Low, Yu Heng Kwan, Benjamin Alan Goldstein, David Bruce Matchar, Bibhas Chakraborty, Nan Liu

**Affiliations:** 1 Programme in Health Services and Systems Research, Duke–National University of Singapore Medical School, Singapore; 2 Department of Emergency Medicine, Singapore General Hospital, Singapore; 3 Department of Family Medicine and Continuing Care, Singapore General Hospital, Singapore; 4 Department of Pharmacy, Faculty of Science, National University of Singapore, Singapore; 5 Department of Biostatistics and Bioinformatics, Duke University, Durham, North Carolina; 6 Duke University Medical Center, Duke University, Durham, North Carolina; 7 Department of Statistics and Data Science, National University of Singapore, Singapore; 8 Health Service Research Centre, Singapore Health Services, Singapore; 9 Institute of Data Science, National University of Singapore, Singapore

## Abstract

**Importance:**

Triage in the emergency department (ED) is a complex clinical judgment based on the tacit understanding of the patient’s likelihood of survival, availability of medical resources, and local practices. Although a scoring tool could be valuable in risk stratification, currently available scores have demonstrated limitations.

**Objectives:**

To develop an interpretable machine learning tool based on a parsimonious list of variables available at ED triage; provide a simple, early, and accurate estimate of patients’ risk of death; and evaluate the tool’s predictive accuracy compared with several established clinical scores.

**Design, Setting, and Participants:**

This single-site, retrospective cohort study assessed all ED patients between January 1, 2009, and December 31, 2016, who were subsequently admitted to a tertiary hospital in Singapore. The Score for Emergency Risk Prediction (SERP) tool was derived using a machine learning framework. To estimate mortality outcomes after emergency admissions, SERP was compared with several triage systems, including Patient Acuity Category Scale, Modified Early Warning Score, National Early Warning Score, Cardiac Arrest Risk Triage, Rapid Acute Physiology Score, and Rapid Emergency Medicine Score. The initial analyses were completed in October 2020, and additional analyses were conducted in May 2021.

**Main Outcomes and Measures:**

Three SERP scores, namely SERP-2d, SERP-7d, and SERP-30d, were developed using the primary outcomes of interest of 2-, 7-, and 30-day mortality, respectively. Secondary outcomes included 3-day mortality and inpatient mortality. The SERP’s predictive power was measured using the area under the curve in the receiver operating characteristic analysis.

**Results:**

The study included 224 666 ED episodes in the model training cohort (mean [SD] patient age, 63.60 [16.90] years; 113 426 [50.5%] female), 56 167 episodes in the validation cohort (mean [SD] patient age, 63.58 [16.87] years; 28 427 [50.6%] female), and 42 676 episodes in the testing cohort (mean [SD] patient age, 64.85 [16.80] years; 21 556 [50.5%] female). The mortality rates in the training cohort were 0.8% at 2 days, 2.2% at 7 days, and 5.9% at 30 days. In the testing cohort, the areas under the curve of SERP-30d were 0.821 (95% CI, 0.796-0.847) for 2-day mortality, 0.826 (95% CI, 0.811-0.841) for 7-day mortality, and 0.823 (95% CI, 0.814-0.832) for 30-day mortality and outperformed several benchmark scores.

**Conclusions and Relevance:**

In this retrospective cohort study, SERP had better prediction performance than existing triage scores while maintaining easy implementation and ease of ascertainment in the ED. It has the potential to be widely applied and validated in different circumstances and health care settings.

## Key Points

**Question:**

How does an interpretable machine learning triage tool for estimating mortality perform in a cohort of individuals admitted to the hospital from the emergency department compared with other clinical scores?

**Findings:**

In this cohort study, the parsimonious and point-based Score for Emergency Risk Prediction was more accurate in identifying patients who died within 2, 7, or 30 days of admissions than other point-based clinical scores.

**Meaning:**

These results suggest that the Score for Emergency Risk Prediction tool shows promise for triaging patients admitted from the emergency department according to mortality risk.

## Teaser

This cohort study examines the performance of an interpretable machine learning triage tool in estimating mortality in individuals admitted to the hospital from the emergency department.

## Introduction

Triage in the emergency department (ED) for admission and appropriate level of hospital care is a complex clinical judgment based on the tacit understanding of the patient’s likely short-term course, availability of medical resources, and local practices.^1,2^ Besides triage categories, early warning scores are also used to identify patients at risk of having adverse events. One such example is the Cardiac Risk Assessment Triage (CART) score,^3^ which calculates a score based on a patient’s vital signs, indicating their risk for cardiac arrest, subsequent transfer to the intensive care unit, and mortality.^4^

To date, few studies^5,6,7,8^ have investigated variables of short-term and long-term mortality among the general ED population, using the limited data available at the point of triage. Most ED-specific scores are targeted toward specific conditions, such as the quick Sepsis-Related Organ Failure Assessment for infection and sepsis,^5,6^ CART for cardiac conditions, or Predicting Mortality in the Emergency Department for elderly populations.^7,8^ Several general purpose scores have been adopted by the ED, such as the Modified Early Warning Score (MEWS) and Acute Physiology and Chronic Health Evaluation (APACHE) II score. However, MEWS has only moderate predictive capabilities, with an area under the curve (AUC) of 0.71,^9^ and APACHE II requires laboratory variables unavailable at the point of triage.^10^ In the fast-paced ED environment, a scoring tool needs to be accurate and straightforward.

To address the need for a risk tool appropriate to the ED workflow, we developed the Score for Emergency Risk Prediction (SERP) using a general-purpose machine learning–based scoring framework named AutoScore.^11^ The resulting tool was compared in a test set to the current triage system used in Singapore, the Patient Acuity Category Scale (PACS),^12^ and several established early warning or triage scores.

## Methods

### Study Design and Setting

We performed a retrospective cohort study of patients seen in the ED of Singapore General Hospital (SGH). Singapore is a city-state in Southeast Asia with a rapidly aging society^13^; currently, approximately 1 in 5 Singaporeans are 60 years or older.^14^ The SGH is the largest and oldest public tertiary hospital in Singapore. The SGH ED receives more than 120 000 visits and has 36 000 inpatient admissions annually. The electronic health record (EHR) data were obtained from Singapore Health Services and analyzed. This study was approved by Singapore Health Services’ Centralized Institutional Review Board, and a waiver of consent was granted for EHR data collection and analysis because of the retrospective nature of the study. All data were deidentified. This study followed the Strengthening the Reporting of Observational Studies in Epidemiology (STROBE) reporting guideline.^15^

### Study Population

All patients visiting the SGH ED from January 1, 2009, until December 31, 2016, who were subsequently admitted, were included. We denote these included episodes as emergency admissions. Patients younger than 21 years were excluded. We also excluded noncitizen patients who might not have complete medical records. These admission episodes from January 1, 2009, to December 31, 2015, were randomly split into 2 nonoverlapping cohorts: a training cohort (80%) and a validation cohort (20%). The admission episodes in 2016 were assigned to the testing cohort. This sequential testing design was chosen to be more consistent with future application scenarios and evaluate whether population shift would influence the model’s performance.

### Outcome

The primary outcomes used to develop and test the tool were 2-, 7-, and 30-day mortality, defined as deaths within 2, 7, and 30 days after emergency admission, respectively. Three SERP scores, namely SERP-2d, SERP-7d, and SERP-30d, were developed using the corresponding primary outcome. We also tested the performance of those clinical scores on the secondary outcomes, including inpatient mortality, defined as deaths in the hospital, and 3-day mortality, defined as deaths within 72 hours after the time of admission. Death records were obtained from the national death registry and were matched to specific patients in the EHR.

### Data Collection and Candidate Variables

We extracted data from the hospital’s EHR through the SingHealth Electronic Health Intelligence System. Patient details were deidentified, complying with Health Insurance Portability and Accountability Act regulations. Comorbidities were obtained from hospital diagnosis and discharge records in the preceding 5 years before patients’ index emergency admissions. They were extracted from the *International Classification of Diseases, Ninth Revision *(*ICD-9*) and *International Statistical Classification of Diseases and Related Health Problems, Tenth Revision *(*ICD-10*),^16^ globally used diagnostic tools for epidemiology and clinical purposes. We preselected candidate variables available in the ED before hospital admission to ensure that SERP was clinically useful and valid for early risk stratification of patients in the ED. Candidate variables included demographic characteristics, administrative variables, medical history in the preceding year, vital signs, and comorbidities. The list of candidate variables is given in the eTable 1 in the Supplement. Comorbidity variables were defined according to the Charlson Comorbidity Index. We used the algorithms developed and updated by Quan et al^17^ for the linkage between Charlson Comorbidity Index and *ICD-9*/*ICD-10* codes.

### Statistical Analysis

The data were analyzed using R software, version 3.5.3 (R Foundation for Statistical Computing). The initial analyses were completed in October 2020, and additional analyses were conducted in May 2021. Baseline characteristics of the study population were analyzed on all 3 cohorts (training, validation, and testing). In the descriptive summaries, numbers (percentages) were reported for categorical variables. For continuous variables, means (SDs) were reported. During the analysis, the value for a vital sign would be considered as an outlier and set to missing if it were beyond the plausible physiologic ranges based on clinical knowledge. For example, any value of vital signs below 0, heart rate above 300/min, respiration rate above 50/min, systolic blood pressure above 300 mm Hg, diastolic blood pressure above 180 mm Hg, or oxygen saturation as measured by pulse oximetry above 100% was deemed an outlier. Subsequently, all missing values were imputed using the median value of the training cohort.

We implemented the AutoScore,^11^ a machine learning–based clinical score generation algorithm, to derive the SERP scoring models. AutoScore combines machine learning and logistic regression, integrates multiple modules of data manipulation, and automates the development of parsimonious sparse-score risk models for predefined outcomes. In addition, it enables users to build interpretable clinical scores quickly and seamlessly, which can be easily implemented and validated in clinical practice. The training cohort was used for the generation of the tentative SERP models using AutoScore framework. The validation cohort was used to evaluate multiple candidate SERP models for parameter tuning and model selection. Then, we calculated the performance metrics of the final SERP model based on the testing cohort. Finally, we used the primary outcomes for model derivation and applied primary and secondary outcomes for model evaluation. The implementation details and methodologic descriptions are elaborated in eFigure 1 and the eMethods in the Supplement.

After model derivation, the predictive performance of the final SERP scores was reported based on the testing cohort, where bootstrapped samples were applied to calculate 95% CIs. Each of the SERP breakdowns was allocated a score that reflected the magnitude of disturbance to each variable. The individual scores were then summed to derive the aggregated SERP score for risk stratification of outcomes. The predictive power of SERP was measured using the AUC in the receiver operating characteristic (ROC) analysis. Sensitivity, specificity, positive predictive value, and negative predictive value were calculated under the optimal threshold, defined as the point nearest to the upper-left corner of the ROC curve. The metrics calculated under different thresholds were also compared to evaluate predictive performance. By using the same testing cohort, we compared the 3 SERP scores with PACS, MEWS,^18^ the National Early Warning Score (NEWS),^19^ CART,^3^ the Rapid Acute Physiology Score (RAPS),^20^ and the Rapid Emergency Medicine Score (REMS),^21^ in estimating multiple mortality outcomes in this study.

## Results

### Baseline Characteristics of the Study Cohort

**Figure 1.**
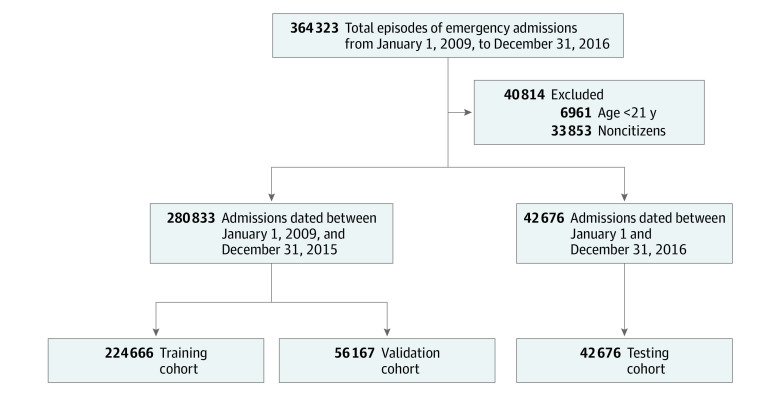
Flow of the Study Cohort Formation

Between January 1, 2009, and December 31, 2015, a total of 280 833 individual admission episodes were assessed, including 224 666 in the training cohort (mean [SD] patient age, 63.60 [16.90] years; 113 426 [50.5%] female) and 56 167 in the validation cohort (mean [SD] patient age, 63.58 [16.87] years; 28 427 [50.6%] female). In addition, 42 676 admission episodes in the year 2016 were included in the testing cohort (mean [SD] patient age, 64.85 [16.80] years; 21 556 [50.5%] female) (Figure 1). The mortality rates observed in the training cohort were 0.8% at 2 days, 2.2% at 7 days, and 5.9% at 30 days. The ethnic compositions were similar to the population norm (74.3% for Chinese, 12.9% for Malay, 10.0% for Indian, and 2.8% for others). A total of 39 548 episodes (17.6%) were triaged as PACS 1, and 128 644 episodes (57.3%) were triaged as PACS 2. Table 1 indicates that patient characteristics in the training and validation cohorts were similar in terms of age, sex, racial and ethnic compositions, and other characteristics. Compared with those in the training and validation cohorts, patients in the testing cohort were slightly older and had a higher risk of triage to PACS 1, with more people having comorbidities of dementia, diabetes, and kidney diseases. The patients in the testing cohort also had marginally lower mortality rates and higher numbers of emergency admissions or operations in the past year. This difference likely reflects the population shift and improvements in health care over time.

**Table 1.** Baseline Characteristics of the Study Cohort^a^

Characteristic	Training (n = 224 666)	Validation (n = 56 167)	Testing (n = 42 676)
Age, mean (SD), y	63.60 (16.90)	63.58 (16.87)	64.85 (16.80)
Sex			
Male	111 240 (49.5)	27 740 (49.4)	21 120 (49.5)
Female	113 426 (50.5)	28 427 (50.6)	21 556 (50.5)
Race/ethnicity			
Chinese	167 004 (74.3)	41 765 (74.4)	31 441 (73.7)
Indian	22 403 (10.0)	5592 (10.0)	4440 (10.4)
Malay	29 040 (12.9)	7213 (12.8)	5465 (12.8)
Other	6219 (2.8)	1597 (2.8)	1330 (3.1)
PACS triage categories			
P1	39 548 (17.6)	9823 (17.5)	9913 (23.2)
P2	128 644 (57.3)	32 058 (57.1)	22 885 (53.6)
P3 and P4	56 474 (25.1)	14 286 (25.4)	9878 (23.1)
Shift time			
8 am to 4 pm	113 758 (50.6)	28 461 (50.7)	21 870 (51.2)
4 pm to midnight	84 503 (37.6)	21 050 (37.5)	15 907 (37.3)
Midnight to 8 am	26 405 (11.8)	6656 (11.9)	4899 (11.5)
Day of week			
Friday	31 553 (14.0)	7893 (14.1)	5839 (13.7)
Monday	37 703 (16.8)	9581 (17.1)	7139 (16.7)
Weekend	57 785 (25.7)	14 283 (25.4)	10 901 (25.5)
Midweek	97 625 (43.5)	24 410 (43.5)	18 797 (44.0)
Vital signs, mean (SD)			
Pulse, /min	81.57 (16.41)	81.62 (16.37)	85.95 (18.36)
Respiration, /min	17.80 (1.57)	17.80 (1.59)	18.23 (2.04)
Spo_2_, %	98.12 (2.84)	98.12 (2.70)	97.34 (4.18)
Blood pressure, mm Hg			
Diastolic	70.99 (13.23)	71.01 (13.20)	72.36 (13.95)
Systolic	133.77 (24.49)	133.80 (24.58)	137.73 (27.87)
Comorbidities			
Myocardial infarction	14 927 (6.6)	3801 (6.8)	2841 (6.7)
Congestive heart failure	28 511 (12.7)	7136 (12.7)	4897 (11.5)
Peripheral vascular disease	14 531 (6.5)	3539 (6.3)	2541 (6.0)
Stroke	32 993 (14.7)	8062 (14.4)	5062 (11.9)
Dementia	6901 (3.1)	1699 (3.0)	1515 (3.6)
Chronic pulmonary disease	24 275 (10.8)	6138 (10.9)	3912 (9.2)
Rheumatoid disease	3341 (1.5)	881 (1.6)	615 (1.4)
Peptic ulcer disease	9879 (4.4)	2505 (4.5)	1362 (3.2)
Diabetes			
None	145 889 (64.9)	36 457 (64.9)	27 204 (63.7)
Diabetes without chronic complications	24 268 (10.8)	6064 (10.8)	1247 (2.9)
Diabetes with complications	54 509 (24.3)	13 646 (24.3)	14 225 (33.3)
Hemiplegia or paraplegia	14 545 (6.5)	3609 (6.4)	1880 (4.4)
Kidney disease	49 884 (22.2)	12 483 (22.2)	10 377 (24.3)
Cancer			
None	185 121 (82.4)	46 251 (82.3)	35 374 (82.9)
Local tumor, leukemia, and lymphoma	20 838 (9.3)	5136 (9.1)	3613 (8.5)
Metastatic solid tumor	18 707 (8.3)	4780 (8.5)	3689 (8.6)
Liver disease			
None	209 865 (93.4)	52 562 (93.6)	39 704 (93.0)
Mild liver disease	11 112 (4.9)	2676 (4.8)	2156 (5.1)
Severe liver disease	3689 (1.6)	929 (1.7)	816 (1.9)
Health care use, mean (SD)			
Emergency admissions in the past year	1.05 (2.35)	1.05 (2.35)	1.12 (2.51)
Operations in the past year	0.20 (0.72)	0.20 (0.72)	0.28 (0.94)
ICU admissions in the past year	0.03 (0.26)	0.02 (0.26)	0.03 (0.29)
HD admissions in the past year	0.10 (0.51)	0.10 (0.51)	0.08 (0.45)
Mortality-related outcomes			
2 d	1801 (0.8)	449 (0.8)	295 (0.7)
3 d	2464 (1.1)	622 (1.1)	416 (1.0)
7 d	4888 (2.2)	1241 (2.2)	779 (1.8)
14 d	8040 (3.6)	2009 (3.6)	1349 (3.2)
Inpatient	8616 (3.8)	2151 (3.8)	1515 (3.6)
30 d	13 244 (5.9)	3285 (5.8)	2310 (5.4)

Abbreviations: HD, high-dependency; ICU, intensive care unit; PACS, Patient Acuity Category Scale; Spo_2_, oxygen saturation as measured by pulse oximetry.

^a^ Data are presented as number (percentage) of patients unless otherwise indicated.

### Selected Variables and SERP Score

AutoScore was used to select the most discriminative variables from all 26 candidate variables (eTable 1 in the Supplement). Parsimony plots (ie, model performance vs complexity) based on the validation set were used for determining the choice of variables (eFigure 2 in the Supplement). We chose 6 variables as the parsimonious choice for SERP-2d and SERP-30d, whereas SERP-7d with 5 variables achieved a good balance in the parsimony plot. Five variables were chosen by all 3 SERP scores, including age, heart rate, respiration rate, diastolic blood pressure, and systolic blood pressure. These selected variables highlighted the importance of vital signs in risk-triaging patients in emergency settings. As seen from eFigure 2 in the Supplement, when more variables were added to the scoring model, the performance was not markedly improved.

The SERP scores derived based on primary outcomes (2-, 7-, and 30-day mortality) were tabulated in Table 2. All 3 scores summed from their included variables ranged from 0 to approximately 60. We used the testing cohort to evaluate the performance of the SERP scores. eFigure 3 in the Supplement depicts the distribution of episodes at different score intervals, which had near-normal distribution. For SERP-2d and SERP-30d, most patients had a risk score between 16 and 24, and few patients had scores under 9 or above 40. As seen in eFigure 4 in the Supplement, the observed mortality rate increased as our risk scores increased in the testing cohort. In terms of different components of SERP, when age was younger than 30 years, its corresponding risk (quantified as points) was the lowest; when age was older than 80 years, the risk was the highest. Likewise, when a reported diastolic blood pressure was between 50 and 94 mm Hg, the corresponding risk was the lowest, and when it was lower than 49 mm Hg, the risk was the highest. Thus, SERP scores had varying points for each component according to the outcomes of interest.

**Table 2.** Three Versions of the SERP Derived From the Primary Outcomes

Variable	SERP scores		
	SERP-2d	SERP-7d	SERP-30d
Age, y			
<30	0	0	0
30-49	9	10	8
50-79	13	17	14
≥80	17	21	19
Heart rate, /min			
<60	3	2	1
60-69	0	0	0
70-94	3	4	2
95-109	6	8	6
≥110	10	12	9
Respiration rate, /min			
<16	11	10	8
16-19	0	0	0
≥20	7	6	6
Blood pressure, mm Hg			
Systolic			
<100	10	12	8
100-114	4	6	5
115-149	1	1	2
≥150	0	0	0
Diastolic			
<50	5	4	3
50-94	0	0	0
≥95	1	2	2
Spo_2_, %			
<90	7	NA	NA
90-94	5	NA	NA
≥95	0	NA	NA
Cancer history			
None	NA	NA	0
Local tumor, leukemia, and lymphoma	NA	NA	6
Metastatic solid tumor	NA	NA	14

Abbreviations: NA, not applicable; SERP, Score for Emergency Risk Prediction; Spo_2_, oxygen saturation as measured by pulse oximetry.

### Performance Evaluation

**Figure 2.**
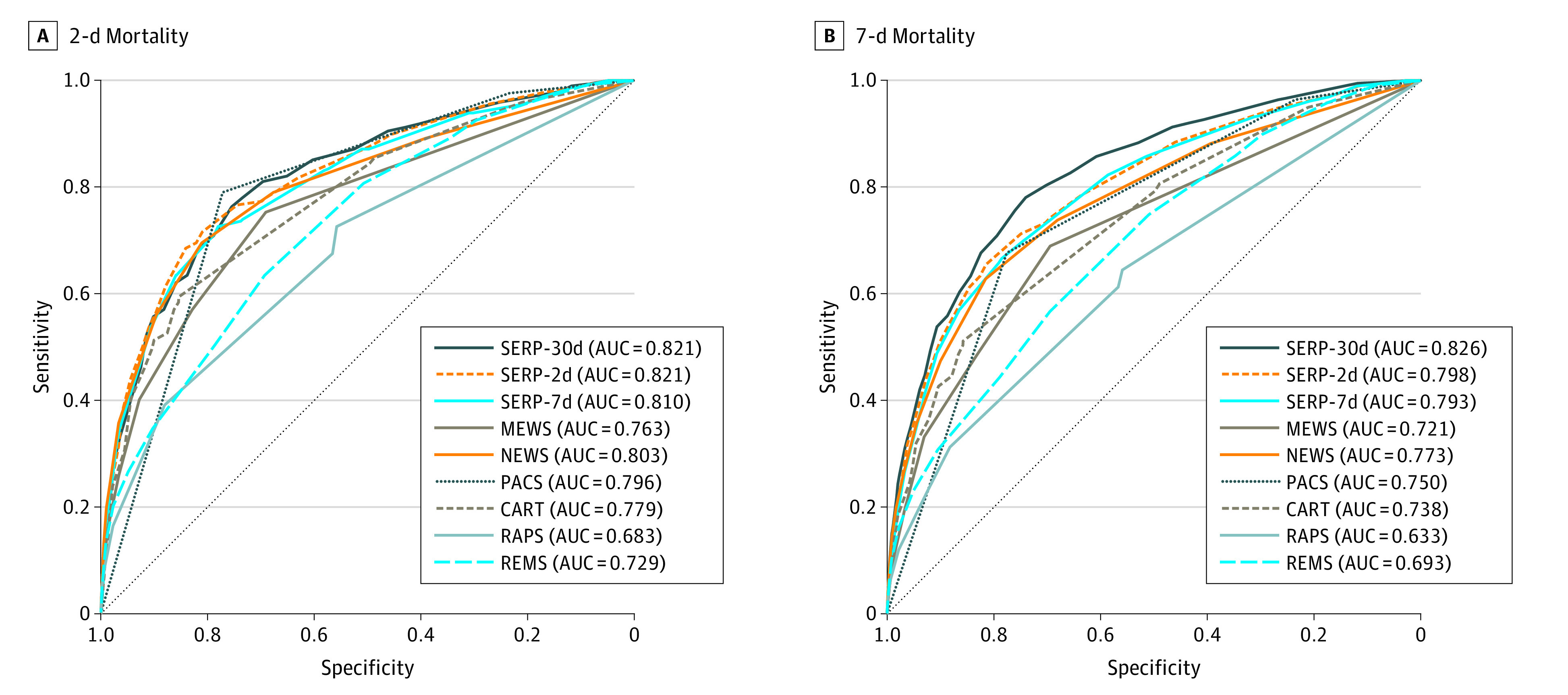
Receiver Operating Characteristic Curves of Score for Emergency Risk Prediction (SERP) Scores and Other Benchmark Clinical Scores for 2- and 7-Day Mortality AUC indicates area under the curve; CART, Cardiac Arrest Risk Triage; MEWS, Modified Early Warning Score; NEWS, National Early Warning Score; PACS, Singapore-based Patient Acuity Category Scale; RAPS, Rapid Acute Physiology Score; REMS, Rapid Emergency Medicine Score.

The performance of the SERP scores and other clinical scores as assessed by ROC analysis in the testing cohort are reported in Table 3 and Figure 2. SERP had promising discriminatory capability in estimating all mortality-related outcomes. The SERP-30d achieved the best performance for short-term and long-term mortality prediction, with an AUC of 0.821 (95% CI, 0.796-0.847) for 2-day mortality, an AUC of 0.826 (95% CI, 0.811-0.841) for 7-day mortality, an AUC of 0.823 (95% CI, 0.814-0.832) for 30-day mortality, and an AUC of 0.810 (95% CI, 0.799-0.821) for inpatient mortality. eTables 2 and 3 in the Supplement summarize the predictive performance of the SERP scores and their comparators on 30- and 2-day mortality risk estimation, respectively.

**Table 3.** Comparison of AUC Values Achieved by Different Triage Scores on the Testing Cohort

Score	AUC value (95% CI) by mortality				
	2 d	3 d	7 d	Inpatient	30 d
SERP-2d	0.821 (0.796-0.847)	0.815 (0.793-0.837)	0.798 (0.781-0.814)	0.769 (0.757-0.781)	0.754 (0.744-0.765)
SERP-7d	0.810 (0.783-0.837)	0.805 (0.783-0.828)	0.793 (0.776-0.809)	0.765 (0.753-0.777)	0.754 (0.744-0.764)
SERP-30d	0.821 (0.796-0.847)	0.824 (0.804-0.845)	0.826 (0.811-0.841)	0.810 (0.799-0.821)	0.823 (0.814-0.832)
CART	0.779 (0.751-0.807)	0.769 (0.745-0.793)	0.738 (0.720-0.756)	0.704 (0.691-0.717)	0.700 (0.689-0.711)
PACS	0.796 (0.775-0.817)	0.778 (0.758-0.797)	0.750 (0.735-0.765)	0.703 (0.691-0.715)	0.680 (0.670-0.690)
MEWS	0.763 (0.734-0.792)	0.750 (0.725-0.774)	0.721 (0.702-0.739)	0.680 (0.667-0.694)	0.663 (0.652-0.674)
NEWS	0.803 (0.774-0.832)	0.792 (0.767-0.817)	0.773 (0.755-0.791)	0.734 (0.720-0.747)	0.711 (0.700-0.723)
RAPS	0.683 (0.652-0.715)	0.674 (0.647-0.700)	0.633 (0.613-0.653)	0.594 (0.580-0.608)	0.580 (0.568-0.591)
REMS	0.729 (0.701-0.758)	0.723 (0.698-0.748)	0.693 (0.674-0.712)	0.669 (0.656-0.682)	0.659 (0.648-0.670)

Abbreviations: AUC, area under the curve; CART, Cardiac Arrest Risk Triage; MEWS, Modified Early Warning Score; NEWS, National Early Warning Score; PACS, Patient Acuity Category Scale; RAPS, Rapid Acute Physiology Score; REMS, Rapid Emergency Medicine Score; SERP, Score for Emergency Risk Prediction.

## Discussion

This cohort study developed parsimonious and point-based SERP scores based on 2-, 7-, and 30-day mortality for risk-stratifying patients after emergency admissions. SERP scores were more accurate in identifying patients who died during short- or long-term care than other point-based clinical tools (ie, PACS, NEWS, MEWS, CART, RAPS, and REMS). A previous study^22^ developed a model for inpatient mortality using variables including basic demographic, administrative, and clinical information acquired in the ED. Despite the model showing good discriminative performance, the need to use a computer with 19 variables to calculate a score limited its applicability and interpretability. Instead, SERP is an additive, point-based triage tool, making it simple, quick to calculate, transparent, and interpretable. Moreover, SERP has the advantage of easy implementation, enabling its wide application in different real-world circumstances.

Among the 3 SERP scores, SERP-30d achieved satisfactory performance on short-term (eg, 2- or 3-day) mortality and relatively long-term (eg, 30-day) mortality risk estimation. Several possible reasons exist for SERP-30d to excel. The 2-day mortality rate in our cohort was as low as 0.8%. Thus, SERP-2d was developed based on highly imbalanced data, for which the abundance of samples from the majority class (survival group) could overwhelm the minority class in predictive modeling. As a comparison, 30-day mortality contained all 2- or 7-day mortality cases and was more prevalent at a rate of 5.9%, making the SERP-30d score more reliable and accurate. Our results reaffirmed the value of 30-day mortality as an essential indicator for the ED.^23,24,25^ Besides vital signs, the SERP-30d score included comorbidities, the importance of which has been demonstrated in several studies.^26,27,28,29^ For example, Chu et al^26^ reported the contribution of patient comorbidity to short- and long-term mortality. Among all 3 SERP scores, age was selected as a key variable through a data-driven process, which aligns with the evidence on the vital role of age among ED patients.^27,28,29^

The SERP scores could provide an objective measure during ED triage to estimate a patient’s mortality risk. Although physicians can generally ascertain the severity of a patient’s acute condition and the threat to life, their decisions are often subjective and depend on an individual’s experience and knowledge. In a study^30^ of elderly patients, although physicians could estimate the 30-day mortality during the consultation, they missed 4 of every 5 deaths, with a sensitivity of 20% only. Like the Emergency Severity Index,^31^ some triage scores may achieve better performance in risk estimation but require some subjective variables. Some recent studies^32,33,34^ highlighted the role of data-driven, objective clinical decision tools to help physicians rethink and reassess the triage process in the ED. Because our SERP scores only comprise objective elements, they can be easily computed by trained medical assistants or integrated into an existing hospital EHR, without the need for professional medical personnel. Therefore, one can rapidly estimate a patient’s risk of death without adversely affecting ED workloads, which is important in the fast-paced ED environment and other heterogenous emergency care systems run by generalists rather than emergency medicine specialists. Given our tool’s purpose as an adjunct to clinical acumen during the consultation, such a risk stratification tool would conceivably be used when a physician plans to admit a patient and considers the level of service that might be appropriate for that individual.

Ultimately, the most important unanswered question is whether SERP can improve outcomes in actual clinical practice. To address this, prospective studies are needed to validate its real-world predictive capabilities and determine appropriate thresholds to stratify the ED population into various risk categories. In addition, given the strength of SERP as a simple and interpretable scoring tool, further assessments could be performed to evaluate the more intangible aspects of score implementation.^35,36^ Such measures would include determining SERP’s long-term sustainability, overall cost-effectiveness, and physician-perceived acceptability. These future assessments might lend credence to SERP as an effective and accurate tool for decision-making within the ED.

### Strengths and Limitations

This study has several strengths. First, machine learning–based variable selection by AutoScore^11^ can efficiently filter out redundant information to achieve a sparse solution. Sanchez-Pinto et al^37^ also suggested that variable selection plays an essential role in reducing the complexity of prediction models without compromising their accuracy, especially when facing a large number of candidate features extracted from EHRs.^38^ Likewise, Liu et al^39^ demonstrated that more variables did not necessarily lead to better prediction of adverse cardiac events. The second strength of SERP is the size of the data set that was used to derive the risk scores. This data set is one of the largest used to generate a point-based triage model, with a cohort of more than 300 000 emergency admissions during 8 years, obtained from a large tertiary hospital. Third, the SERP scores consistently performed well in the testing cohort, even with changes in patient characteristics, outcome prevalence, and clinical practices amid the continuously evolving clinical environment.^40^

This study also has several limitations. First, the data set used in this study was based on EHR data of routinely collected variables. Thus, some variables, such as socioeconomic status, were not used in SERP score development. Second, because this was a single-center study at a tertiary hospital, the performance of SERP scores may vary in different settings. Third, our ED cohort accounted for only ED admissions, which might influence score generalizability when applying the scores to a general ED population.

## Conclusions

SERP is a parsimonious and point-based scoring tool for triaging patients in the ED. In this cohort study, SERP performed better in comparison with existing triage scores and has the advantage of easy implementation and ease of ascertainment at ED presentation. SERP scores have the potential to be widely used and validated in different circumstances and health care settings. Following the clinical application of SERP in ED triage, more tailored scores can be derived in various clinical areas through the machine learning–based AutoScore framework in the future.

## Notes

Supplement. **eTable 1.** List of Candidate Variables and Their Definitions **eTable 2.** Varying Cutoffs of Predicted Risk Based on the SERP-30d, MEWS, NEWS, CART, RAPS, and REMS, the Proportion of Patients Stratified for 30-Day Mortality, and the Corresponding Sensitivity, Specificity, Positive and Negative Predictive Values on the Testing Cohort **eTable 3.** Varying Cutoffs of Predicted Risk Based on the SERP-2d, SERP-7d, SERP-30d, MEWS, NEWS, CART, RAPS, and REMS, the Proportion of Patients Stratified for 2-Day Mortality, and the Corresponding Sensitivity and Specificity on the Testing Cohort **eFigure 1.** Flowchart of the AutoScore Framework **eFigure 2.** Parsimony Plot of the Number of Variables Vs AUC Values for (A) SERP-2d, (B) SERP-7d, and (C) SERP-30d on the Validation Cohort **eFigure 3.** Number of Cases Versus Score Value on the Testing Cohort for (A) SERP-2d and (B) SERP-30d **eFigure 4.** Observed Mortality Rate Versus Score Value on the Testing Cohort for (A) SERP-2d and (B) SERP-30d **eMethods.** Description of the AutoScore Method

## References

[1] Hinson JS, Martinez DA, Cabral S, . Triage performance in emergency medicine: a systematic review. Ann Emerg Med. 2019;74(1):140-152. doi:10.1016/j.annemergmed.2018.09.022 30470513

[2] Htay T, Aung K. Review: some ED triage systems better predict ED mortality than in-hospital mortality or hospitalization. Ann Intern Med. 2019;170(8):JC47. doi:10.7326/ACPJ201904160-047 30986837

[3] Churpek MM, Yuen TC, Park SY, Meltzer DO, Hall JB, Edelson DP. Derivation of a cardiac arrest prediction model using ward vital signs. Crit Care Med. 2012;40(7):2102-2108. doi:10.1097/CCM.0b013e318250aa5a 22584764 PMC3378796

[4] Fernandes M, Vieira SM, Leite F, Palos C, Finkelstein S, Sousa JMC. Clinical decision support systems for triage in the emergency department using intelligent systems: a review. Artif Intell Med. 2020;102:101762. doi:10.1016/j.artmed.2019.101762 31980099

[5] Williams JM, Greenslade JH, Chu K, Brown AFT, Lipman J. Severity scores in emergency department patients with presumed infection: a prospective validation study. Crit Care Med. 2016;44(3):539-547. doi:10.1097/CCM.0000000000001427 26901543

[6] Xia Y, Zou L, Li D, . The ability of an improved qSOFA score to predict acute sepsis severity and prognosis among adult patients. Medicine (Baltimore). 2020;99(5):e18942. doi:10.1097/MD.0000000000018942 32000414 PMC7004789

[7] Moman RN, Loprinzi Brauer CE, Kelsey KM, Havyer RD, Lohse CM, Bellolio MF. PREDICTing Mortality in the Emergency Department: external validation and derivation of a clinical prediction tool. Acad Emerg Med. 2017;24(7):822-831. doi:10.1111/acem.13197 28401622

[8] Cardona M, O’Sullivan M, Lewis ET, . Prospective validation of a checklist to predict short-term death in older patients after emergency department admission in Australia and Ireland. Acad Emerg Med. 2019;26(6):610-620. doi:10.1111/acem.13664 30428145 PMC6619350

[9] Eick C, Rizas KD, Meyer-Zürn CS, . Autonomic nervous system activity as risk predictor in the medical emergency department: a prospective cohort study. Crit Care Med. 2015;43(5):1079-1086. doi:10.1097/CCM.0000000000000922 25738854

[10] Olsson T, Lind L. Comparison of the rapid emergency medicine score and APACHE II in nonsurgical emergency department patients. *Acad Emerg Med**.* 2003;10(10):1040-1048. doi:10.1111/j.1553-2712.2003.tb00572.x 14525735

[11] Xie F, Chakraborty B, Ong MEH, Goldstein BA, Liu N. AutoScore: a machine learning–based automatic clinical score generator and its application to mortality prediction using electronic health records. JMIR Med Inform. 2020;8(10):e21798. doi:10.2196/21798 33084589 PMC7641783

[12] Fong RY, Glen WSS, Mohamed Jamil AK, Tam WWS, Kowitlawakul Y. Comparison of the emergency severity index versus the patient acuity category scale in an emergency setting. Int Emerg Nurs. 2018;41:13-18. doi:10.1016/j.ienj.2018.05.001 29887281

[13] Malhotra R, Bautista MAC, Müller AM, . The aging of a young nation: population aging in Singapore. Gerontologist. 2019;59(3):401-410. 30517628 10.1093/geront/gny160

[14] Department of Statistics, Ministry of Trade & Industry. Population trends, 2020. Published 2020. Accessed November 1, 2020. https://www.singstat.gov.sg/-/media/files/publications/population/population2020.pdf

[15] von Elm E, Altman DG, Egger M, Pocock SJ, Gøtzsche PC, Vandenbroucke JP; STROBE Initiative. The Strengthening the Reporting of Observational Studies in Epidemiology (STROBE) statement: guidelines for reporting observational studies. Lancet. 2007;370(9596):1453-1457. doi:10.1016/S0140-6736(07)61602-X 18064739

[16] World Health Organization. International Classification of Diseases, Ninth Revision (ICD-9). Geneva, Switzerland: World Health Organization; 1977.

[17] Quan H, Sundararajan V, Halfon P, . Coding algorithms for defining comorbidities in ICD-9-CM and ICD-10 administrative data. Med Care. 2005;43(11):1130-1139. doi:10.1097/01.mlr.0000182534.19832.83 16224307

[18] Subbe CP, Kruger M, Rutherford P, Gemmel L. Validation of a modified Early Warning Score in medical admissions. QJM. 2001;94(10):521-526. doi:10.1093/qjmed/94.10.521 11588210

[19] Royal College of Physician. National early warning score (NEWS) 2: standardising the assessment of acute-illness severity in the NHS. 2017. Accessed November 1, 2021. https://www.rcplondon.ac.uk/projects/outputs/national-early-warning-score-news-2

[20] Rhee KJ, Fisher CJ Jr, Willitis NH. The Rapid Acute Physiology Score. Am J Emerg Med. 1987;5(4):278-282. doi:10.1016/0735-6757(87)90350-0 3593492

[21] Olsson T, Terent A, Lind L. Rapid Emergency Medicine score: a new prognostic tool for in-hospital mortality in nonsurgical emergency department patients. J Intern Med. 2004;255(5):579-587. doi:10.1111/j.1365-2796.2004.01321.x 15078500

[22] Xie F, Liu N, Wu SX, . Novel model for predicting inpatient mortality after emergency admission to hospital in Singapore: retrospective observational study. BMJ Open. 2019;9(9):e031382. doi:10.1136/bmjopen-2019-031382 31558458 PMC6773418

[23] Kaeppeli T, Rueegg M, Dreher-Hummel T, . Validation of the Clinical Frailty Scale for prediction of thirty-day mortality in the emergency department. Ann Emerg Med. 2020;76(3):291-300. doi:10.1016/j.annemergmed.2020.03.028 32336486

[24] Zelis N, Buijs J, de Leeuw PW, van Kuijk SMJ, Stassen PM. A new simplified model for predicting 30-day mortality in older medical emergency department patients: the rise up score. Eur J Intern Med. 2020;77:36-43. doi:10.1016/j.ejim.2020.02.021 32113943

[25] Blomaard LC, Speksnijder C, Lucke JA, . Geriatric screening, triage urgency, and 30-day mortality in older emergency department patients. J Am Geriatr Soc. 2020;68(8):1755-1762. doi:10.1111/jgs.16427 32246476 PMC7497167

[26] Chu YT, Ng YY, Wu SC. Comparison of different comorbidity measures for use with administrative data in predicting short- and long-term mortality. BMC Health Serv Res. 2010;10:140. doi:10.1186/1472-6963-10-140 20507593 PMC2897792

[27] Vilpert S, Monod S, Jaccard Ruedin H, . Differences in triage category, priority level and hospitalization rate between young-old and old-old patients visiting the emergency department. BMC Health Serv Res. 2018;18(1):456. doi:10.1186/s12913-018-3257-9 29907110 PMC6003168

[28] Goodacre S, Turner J, Nicholl J. Prediction of mortality among emergency medical admissions. Emerg Med J. 2006;23(5):372-375. doi:10.1136/emj.2005.028522 16627839 PMC2564087

[29] Parker CA, Liu N, Wu SX, Shen Y, Lam SSW, Ong MEH. Predicting hospital admission at the emergency department triage: a novel prediction model. Am J Emerg Med. 2019;37(8):1498-1504. doi:10.1016/j.ajem.2018.10.060 30413365

[30] Ouchi K, Strout T, Haydar S, . Association of emergency clinicians’ assessment of mortality risk with actual 1-month mortality among older adults admitted to the hospital. JAMA Netw Open. 2019;2(9):e1911139. doi:10.1001/jamanetworkopen.2019.11139 31517962 PMC6745053

[31] Wuerz RC, Milne LW, Eitel DR, Travers D, Gilboy N. Reliability and validity of a new five-level triage instrument. Acad Emerg Med. 2000;7(3):236-242. doi:10.1111/j.1553-2712.2000.tb01066.x 10730830

[32] Miles J, Turner J, Jacques R, Williams J, Mason S. Using machine-learning risk prediction models to triage the acuity of undifferentiated patients entering the emergency care system: a systematic review. Diagn Progn Res. 2020;4(1):16. doi:10.1186/s41512-020-00084-1 33024830 PMC7531169

[33] Liu N, Guo D, Koh ZX, . Heart rate n-variability (HRnV) and its application to risk stratification of chest pain patients in the emergency department. BMC Cardiovasc Disord. 2020;20(1):168. doi:10.1186/s12872-020-01455-8 32276602 PMC7149930

[34] Goto T, Camargo CA Jr, Faridi MK, Freishtat RJ, Hasegawa K. Machine learning-based prediction of clinical outcomes for children during emergency department triage. JAMA Netw Open. 2019;2(1):e186937. doi:10.1001/jamanetworkopen.2018.6937 30646206 PMC6484561

[35] Khadjesari Z, Boufkhed S, Vitoratou S, . Implementation outcome instruments for use in physical healthcare settings: a systematic review. Implement Sci. 2020;15(1):66. doi:10.1186/s13012-020-01027-6 32811517 PMC7433178

[36] Cowley LE, Farewell DM, Maguire S, Kemp AM. Methodological standards for the development and evaluation of clinical prediction rules: a review of the literature. Diagn Progn Res. 2019;3(1):16. doi:10.1186/s41512-019-0060-y 31463368 PMC6704664

[37] Sanchez-Pinto LN, Venable LR, Fahrenbach J, Churpek MM. Comparison of variable selection methods for clinical predictive modeling. Int J Med Inform. 2018;116:10-17. doi:10.1016/j.ijmedinf.2018.05.006 29887230 PMC6003624

[38] Gronsbell J, Minnier J, Yu S, Liao K, Cai T. Automated feature selection of predictors in electronic medical records data. Biometrics. 2019;75(1):268-277. doi:10.1111/biom.12987 30353541

[39] Liu N, Koh ZX, Goh J, . Prediction of adverse cardiac events in emergency department patients with chest pain using machine learning for variable selection. BMC Med Inform Decis Mak. 2014;14:75. doi:10.1186/1472-6947-14-75 25150702 PMC4150554

[40] Davis SE, Greevy RA, Lasko TA, Walsh CG, Matheny ME. Comparison of prediction model performance updating protocols: using a data-driven testing procedure to guide updating. AMIA Annu Symp Proc. 2020;2019:1002-1010. 32308897 PMC7153129

