# Supplementary material for: Development and Assessment of an Interpretable Machine Learning Triage Tool for Estimating Mortality After Emergency Admissions
Source: JAMA Netw Open. 2021 Aug 27;4(8):e2118467. doi: 10.1001/jamanetworkopen.2021.18467 (PMC8397930; doi:10.1001/jamanetworkopen.2021.18467)
Supplement: Supplement. — eTable 1. List of Candidate Variables and Their Definitions eTable 2. Varying Cutoffs of Predicted Risk Based on the SERP-30d, MEWS, NEWS, CART, RAPS, and REMS, the Proportion of Patients Stratified for 30-Day Mortality, and the Corresponding Sensitivity, Specificity, Positive and Negative Predictive Values on the Testing Cohort eTable 3. Varying Cutoffs of Predicted Risk Based on the SERP-2d, SERP-7d, SERP-30d, MEWS, NEWS, CART, RAPS, and REMS, the Proportion of Patients Stratified for 2-Day Mortality, and the Corresponding Sensitivity and Specificity on the Testing Cohort eFigure 1. Flowchart of the AutoScore Framework eFigure 2. Parsimony Plot of the Number of Variables Vs AUC Values for (A) SERP-2d, (B) SERP-7d, and (C) SERP-30d on the Validation Cohort eFigure 3. Number of Cases Versus Score Value on the Testing Cohort for (A) SERP-2d and (B) SERP-30d eFigure 4. Observed Mortality Rate Versus Score Value on the Testing Cohort for (A) SERP-2d and (B) SERP-30d eMethods. Description of the AutoScore Method [file jamanetwopen-e2118467-s001.pdf]

Supplementary Online Content

Xie F, Ong MEH, Liew JNMH, et al. Development and assessment of an interpretable machine learning triage tool for estimating mortality after emergency admissions. *JAMA Netw Open*. 2021;4(8):e2118467. doi:10.1001/jamanetworkopen.2021.18467

eTable 1. List of Candidate Variables and Their Definitions

eTable 2. Varying Cutoffs of Predicted Risk Based on the SERP-30d, MEWS, NEWS, CART, RAPS, and REMS, the Proportion of Patients Stratified for 30-Day Mortality, and the Corresponding Sensitivity, Specificity, Positive and Negative Predictive Values on the Testing Cohort

eTable 3. Varying Cutoffs of Predicted Risk Based on the SERP-2d, SERP-7d, SERP-30d, MEWS, NEWS, CART, RAPS, and REMS, the Proportion of Patients Stratified for 2-Day Mortality, and the Corresponding Sensitivity and Specificity on the Testing Cohort

eFigure 1. Flowchart of the AutoScore Framework

eFigure 2. Parsimony Plot of the Number of Variables Versus AUC Values for (A) SERP-2d, (B) SERP-7d, and (C) SERP-30d on the Validation Cohort

eFigure 3. Number of Cases Versus Score Value on the Testing Cohort for (A) SERP-2d and (B) SERP-30d

eFigure 4. Observed Mortality Rate Versus Score Value on the Testing Cohort for (A) SERP-2d and (B) SERP-30d

eMethods. Description of the AutoScore Method

This supplementary material has been provided by the authors to give readers additional information about their work.

© 2021 Xie F et al. *JAMA Network Open.*

eTable 1. List of Candidate Variables and Their Definitions

Candidate Variable Name	Definition
Demographics & Administrative	
Age	Age in years, specific to the index emergency admission
Gender	Gender identity as identified in official patient identification documents
Day of week	Days of the week, classified either as Monday, midweek (Tuesday, Wednesday, Thursday), Friday, or weekend (Saturday, Sunday)
Shift time	8-hour shift times ranging 08:00 to 16:00, 16:00 to 24:00, and 24:00 to 8:00
Medical history in the preceding year	
Count of emergency admissions last year	Total number of unique hospital admissions from the ED within a time period of one year from the index emergency admission
Count of surgeries last year	Total number of in-hospital surgeries within a time period of one year from the index emergency admission
Count of ICU admissions last year	The total number of Intensive Care Unit (ICU) admissions within a time period of one year from the index emergency admission
Count of HD admissions last year	The total number of High Dependency (HD) admissions within a time period of one year from the index emergency admission
Clinical data	
Pulse	The first measurement of the number of heart beats per minute during the index presentation to the ED. Typically electronically obtained during automated blood pressure cuff measurement.
Respiration	The first measurement of the number of breaths taken per minute during the index presentation to the ED. Typically visually obtained by an experienced triage nurse in most circumstances.
SpO_2	The first measurement of the peripheral capillary oxygen percentage saturation during the index presentation to the ED. Typically electronically obtained using a portable fingertip pulse oximeter.
Diastolic BP	The first measurement of the diastolic blood pressure (BP) during the index presentation to the ED. Typically electronically obtained during automated blood pressure cuff measurement.

© 2021 Xie F et al. *JAMA Network Open.*

Systolic BP	The first measurement of the systolic blood pressure (BP) during the index presentation to the ED. Typically electronically obtained during automated blood pressure cuff measurement.
Comorbidities (ICD-9 and ICD-10 codes were extracted from patient medical records in the preceding five years and matched to their corresponding comorbidities based on prior work by Quan et. al. Comorbidity was deemed to be absent if the representative codes were not identified)	
Myocardial infarction	ICD-9-CM: 410.x, 412.x, or ICD-10: I21.x, I22.x, I25.2
Congestive heart failure	ICD-9-CM: 428.x, or ICD-10: I09.9, I11.0, I13.0, I13.2, I25.5, I42.0, I42.5–I42.9, I43.x, I50.x, P29.0
Peripheral vascular disease	ICD-9-CM: 443.9, 441.x, 785.4, V43.4, or Procedure 38.48, or ICD-10: I70.x, I71.x, I73.1, I73.8, I73.9, I77.1, I79.0, I79.2, K55.1, K55.8, K55.9, Z95.8, Z95.9
Stroke	Eg cerebrovascular accident (CVA) or transient ischemic attack (TIA)<br>ICD-9-CM: 430.x–438.x, or ICD-10: G45.x, G46.x, H34.0, I60.x–I69.x
Dementia	ICD-9-CM: 490.x–505.x, 506.4, or ICD-10: F00.x–F03.x, F05.1, G30.x, G31.1
Chronic pulmonary disease	ICD-9-CM: 290.x, or ICD-10: I27.8, I27.9, J40.x–J47.x, J60.x–J67.x, J68.4, J70.1, J70.3
Rheumatoid disease	ICD-9-CM: 710.0, 710.1, 710.4,714.0–714.2, 714.81, 725.x, or ICD-10: M05.x, M06.x, M31.5, M32.x–M34.x, M35.1, M35.3, M36.0
Peptic ulcer disease	ICD-9-CM: 531.x–534.x, or ICD-10: K25.x–K28.x
Diabetes without chronic complications	ICD-9-CM: 250.0–250.3, 250.7, or ICD-10: E10.0, E10.1, E10.6, E10.8, E10.9, E11.0, E11.1, E11.6, E11.8, E11.9, E12.0, E12.1, E12.6, E12.8, E12.9, E13.0, E13.1, E13.6, E13.8, E13.9, E14.0, E14.1, E14.6, E14.8, E14.9
Diabetes with complications	ICD-9-CM: 250.4–250.6, or ICD-10: E10.2–E10.5, E10.7, E11.2–E11.5, E11.7, E12.2–E12.5, E12.7, E13.2–E13.5, E13.7, E14.2–E14.5, E14.7
Hemiplegia or paraplegia	ICD-9-CM: 344.1, 342.x, or ICD-10: G04.1, G11.4, G80.1, G80.2, G81.x, G82.x, G83.0–G83.4, G83.9
Renal disease	ICD-9-CM: 582.x, 583–583.7, 585.x, 586.x, 588.x, or ICD-10: I12.0, I13.1, N03.2–N03.7, N05.2–N05.7, N18.x, N19.x, N25.0, Z49.0–Z49.2, Z94.0, Z99.2

© 2021 Xie F et al. *JAMA Network Open.*

Local tumor, leukemia, or lymphoma (except malignant neoplasm of skin)	ICD-9-CM: 140.x–172.x, 174.x.–195.8, 200.x–208.x, or ICD-10: C00.x–C26.x, C30.x–C34.x, C37.x–C41.x, C43.x, C45.x–C58.x, C60.x–C76.x, C81.x–C85.x, C88.x, C90.x–C97.x
Metastatic solid tumor	ICD-9-CM:196.x–199.1, or ICD-10:: C77.x–C80.x
Mild liver disease	ICD-9-CM: 571.2, 571.4–571.6, or ICD-10: B18.x, K70.0–K70.3, K70.9, K71.3–K71.5, K71.7, K73.x, K74.x, K76.0, K76.2–K76.4, K76.8, K76.9, Z94.4
Severe liver disease	ICD-9-CM: 456.0–456.21, 572.2–572.8, or ICD-10: I85.0, I85.9, I86.4, I98.2, K70.4, K71.1, K72.1, K72.9, K76.5, K76.6, K76.7
Mortality Outcomes	
Inpatient Mortality	A clinically certified death of an admitted patient that happened during the in-patient hospital stay
2-day mortality	A clinically certified death of an admitted patient that happened 48 hours after emergency admission
3-day mortality	A clinically certified death of an admitted patient that happened 72 hours after emergency admission
7-day mortality	A clinically certified death of an admitted patient that happened seven days after emergency admission
30-day mortality	A clinically certified death of an admitted patient that happened 30 days after emergency admission

ICD-9-CM: International Classification of Diseases, Ninth Revision, Clinical Modification

ICD-10: The International Statistical Classification of Diseases and Related Health Problems 10th Revision

© 2021 Xie F et al. *JAMA Network Open.*

eTable 2. Varying Cutoffs of Predicted Risk Based on the SERP-30d, MEWS, NEWS, CART, RAPS, and REMS, the Proportion of Patients Stratified for 30-Day Mortality, and the Corresponding Sensitivity, Specificity, Positive and Negative Predictive Values on the Testing Cohort

Score cut-off	Predicted Risk	Percentage of patients (%)	Sensitivity (95% CI)	Specificity (95% CI)	PPV (95% CI)	NPV (95% CI)
**SERP-30d**						
≥15	≥1%	88	99.1% (98.7-99.5%)	12.2% (11.9-12.5%)	6.1% (6-6.1%)	99.6% (99.4-99.8%)
≥26[a]	≥5%	27	74.6% (72.9-76.5%)	75.8% (75.4-76.2%)	15% (14.6-15.4%)	98.1% (98-98.3%)
≥31	≥10%	14	56.5% (54.4-58.5%)	88% (87.7-88.3%)	21.2% (20.4-21.9%)	97.2% (97.1-97.4%)
≥37	≥20%	5	30.8% (29-32.8%)	96.5% (96.3-96.6%)	33.2% (31.5-35%)	96.1% (96-96.2%)
≥46	≥50%	1	6.1% (5.2-7.1%)	99.7% (99.7-99.8%)	55.3% (49.4-61%)	94.9% (94.8-94.9%)
**MEWS**						
≥2[a]	≥5%	31	59% (57-61%)	70.3% (69.9-70.8%)	10.2% (9.9-10.6%)	96.8% (96.6-96.9%)
≥4	≥10%	7	23.5% (21.7-25.3%)	93.5% (93.3-93.7%)	17.1% (15.9-18.4%)	95.5% (95.4-95.6%)
≥6	≥20%	1	5.6% (4.7-6.5%)	98.8% (98.7-98.9%)	21.6% (18.3-24.9%)	94.8% (94.8-94.9%)
≥9	≥50%	0	0.4% (0.2-0.7%)	100% (99.9-100%)	33.3% (16.7-51.5%)	94.6% (94.6-94.6%)
**NEWS**						
≥2[a]	≥5%	33	63.5% (61.4-65.4%)	69% (68.6-69.5%)	10.5% (10.2-10.8%)	97.1% (96.9-97.2%)
≥4	≥10%	11	35.2% (33.3-37.1%)	90.7% (90.4-91%)	17.8% (16.9-18.7%)	96.1% (96-96.2%)
≥7	≥20%	2	10.9% (9.7-12.2%)	98.6% (98.4-98.7%)	30% (27.3-32.9%)	95.1% (95-95.1%)
≥10	≥50%	0	2.3% (1.7-2.9%)	99.8% (99.7-99.8%)	37.7% (29.7-45.9%)	94.7% (94.7-94.7%)
**CART**						
≥6 [a]	≥3.8%	52	77.4% (75.6-79.1%)	49.9% (49.4-50.4%)	8.1% (7.9-8.3%)	97.5% (97.3-97.7%)

© 2021 Xie F et al. *JAMA Network Open.*

≥9	≥5%	51	76% (74.2-77.7%)	50.8% (50.3-51.2%)	8.1% (7.9-8.3%)	97.4% (97.2-97.6%)
≥17	≥10%	10	30.7% (28.8-32.6%)	91.4% (91.1-91.7%)	17% (16-17.9%)	95.8% (95.7-95.9%)
≥27	≥20%	2	8.1% (7-9.2%)	98.7% (98.6-98.8%)	26.7% (23.5-29.7%)	94.9% (94.9-95%)
≥43	≥50%	0	1% (0.6-1.4%)	99.9% (99.8-99.9%)	30.6% (20.5-40.9%)	94.6% (94.6-94.7%)
**RAPS**						
≥1	≥5%	44	56.9% (54.9-59%)	56.3% (55.8-56.7%)	6.9% (6.7-7.2%)	95.8% (95.6-96%)
≥5	≥10%	2	6.8% (5.8-7.9%)	97.9% (97.8-98.1%)	15.7% (13.6-18.1%)	94.8% (94.8-94.9%)
≥8	≥20%	0	0.8% (0.5-1.2%)	99.9% (99.9-100%)	45.2% (30-60%)	94.6% (94.6-94.6%)
**REMS**						
≥6	≥5%	50	71.1% (69.2-73%)	51.7% (51.2-52.2%)	7.8% (7.6-8%)	96.9% (96.7-97.1%)
≥9	≥10%	10	22.2% (20.6-24%)	90.8% (90.5-91.1%)	12.2% (11.3-13.1%)	95.3% (95.2-95.4%)
≥13	≥20%	0	3.1% (2.4-3.9%)	99.7% (99.6-99.7%)	34.8% (28.1-41.1%)	94.7% (94.7-94.8%)
≥19	≥50%	0	0.4% (0.2-0.6%)	100% (100-100%)	90.9% (66.7-100%)	94.6% (94.6-94.6%)

SERP, Score for Emergency Risk Prediction; MEWS, Modified Early Warning Score; NEWS, National Early Warning Score; CART, Cardiac Arrest Risk Triage; RAPS, Rapid Acute Physiology Score; REMS, Rapid Emergency Medicine score.

[a] Optimal threshold, defined as the point nearest to the upper-left corner of the ROC curve

© 2021 Xie F et al. *JAMA Network Open.*

eTable 3. Varying Cutoffs of Predicted Risk Based on the SERP-2d, SERP-7d, SERP-30d, MEWS, NEWS, CART, RAPS, and REMS, the Proportion of Patients Stratified for 2-Day Mortality, and the Corresponding Sensitivity and Specificity on the Testing Cohort

Predicted Risk	Score cut-off	Percentage of patients	Sensitivity (95% CI)	Specificity (95% CI)
**SERP-2d**				
≥1%	≥27 [a]	16	68.5% (63.1-73.9%)	84.1% (83.7-84.5%)
≥5%	≥38	2	21.7% (16.9-26.4%)	98.3% (98.2-98.4%)
≥10%	≥43	1	9.8% (6.4-13.2%)	99.5% (99.5-99.6%)
≥20%	≥48	0	4.1% (2-6.4%)	99.9% (99.8-99.9%)
**SERP-7d**				
≥0.7%	≥30 [a]	22	72.5% (67.5-77.3%)	78% (77.6-78.4%)
≥1%	≥32	14	63.4% (58-68.8%)	85.9% (85.5-86.2%)
≥5%	≥43	1	18.3% (13.9-22.7%)	98.7% (98.6-98.8%)
≥10%	≥49	0	3.1% (1.4-5.1%)	99.7% (99.7-99.8%)
≥20%	≥55	0	1% (0-2.4%)	100% (100-100%)
**SERP-30d**				
≥0.6%	≥27 [a]	25	76.3% (71.5-81%)	75.5% (75.1-75.9%)
≥1%	≥30	17	63.4% (58-68.5%)	83.8% (83.5-84.1%)
≥5%	≥42	2	23.4% (18.6-28.1%)	98% (97.9-98.2%)
≥10%	≥48	0	10.2% (6.8-13.6%)	99.6% (99.5-99.7%)
≥20%	≥54	0	1.4% (0.3-2.7%)	99.9% (99.9-100%)
**MEWS**				
≥0.5%	≥2 [a]	31	75.3% (70.2-80%)	69.1% (68.7-69.5%)
≥1%	≥3	17	56.9% (51.2-62.7%)	82.9% (82.5-83.2%)
≥5%	≥6	1	15.9% (11.9-20.3%)	98.7% (98.6-98.8%)
≥10%	≥8	0	3.4% (1.7-5.8%)	99.8% (99.8-99.9%)
≥20%	≥9	0	1.7% (0.3-3.4%)	99.9% (99.9-100%)
≥50%	≥11	0	0.7% (0-1.7%)	100% (100-100%)
**NEWS**				
≥0.7%	≥3 [a]	19	69.5% (64.4-74.6%)	81.1% (80.7-81.5%)
≥1%	≥4	11	55.9% (50.2-61.7%)	89.6% (89.3-89.9%)
≥5%	≥8	1	19.7% (15.3-24.1%)	99% (98.9-99.1%)
≥10%	≥9	1	12.9% (9.2-16.6%)	99.5% (99.4-99.6%)
≥20%	≥11	0	4.1% (2-6.4%)	99.9% (99.8-99.9%)
≥50%	≥14	0	0% (0-0%)	100% (100-100%)
**CART**				
≥0.6%	≥10 [a]	15	59.3% (53.9-64.8%)	85.4% (85.1-85.7%)
≥1%	≥15	10	51.2% (45.4-57%)	90% (89.8-90.3%)
≥5%	≥30	1	14.6% (10.5-18.6%)	98.6% (98.5-98.8%)

© 2021 Xie F et al. *JAMA Network Open.*

≥10%	≥37	0	4.4% (2.4-7.1%)	99.6% (99.6-99.7%)
≥20%	≥44	0	2% (0.7-3.7%)	99.8% (99.8-99.9%)
≥50%	≥58	0	0% (0-0%)	100% (100-100%)
**RAPS**				
≥0.4%	≥1 [a]	44	72.5% (67.5-77.6%)	55.7% (55.2-56.2%)
≥1%	≥3	12	39% (33.6-44.4%)	88.1% (87.8-88.4%)
≥5%	≥7	0	5.1% (3.1-7.8%)	99.8% (99.8-99.8%)
≥10%	≥8	0	3.4% (1.4-5.4%)	99.9% (99.9-100%)
≥20%	≥10	0	1.7% (0.3-3.4%)	100% (100-100%)
≥50%	≥13	0	0% (0-0%)	100% (100-100%)
**REMS**				
≥0.7%	≥7 [a]	31	63.4% (58-69.2%)	69.3% (68.8-69.7%)
≥1%	≥8	21	50.2% (44.4-55.9%)	78.7% (78.3-79.1%)
≥5%	≥13	0	7.8% (5.1-10.8%)	99.6% (99.5-99.6%)
≥10%	≥15	0	3.1% (1.4-5.1%)	99.9% (99.9-99.9%)
≥20%	≥18	0	1.4% (0.3-2.7%)	100% (100-100%)
≥50%	≥22	0	0.7% (0-1.7%)	100% (100-100%)

SERP, Score for Emergency Risk Prediction; MEWS, Modified Early Warning Score; NEWS, National Early Warning Score; CART, Cardiac Arrest Risk Triage; RAPS, Rapid Acute Physiology Score; REMS, Rapid Emergency Medicine score.

[a]Optimal threshold, defined as the point nearest to the upper-left corner of the ROC curve

© 2021 Xie F et al. *JAMA Network Open.*

eFigure 1. Flowchart of the AutoScore Framework

This figure was originally published in JMIR Medical Informatics (http://medinform.jmir.org) under CC-BY license: Xie F, Chakraborty B, Ong MEH, Goldstein BA, Liu N. AutoScore: A Machine Learning–Based Automatic Clinical Score Generator and Its Application to Mortality Prediction Using Electronic Health Records. JMIR Med Inform 2020;8(10):e21798. doi: 10.2196/21798

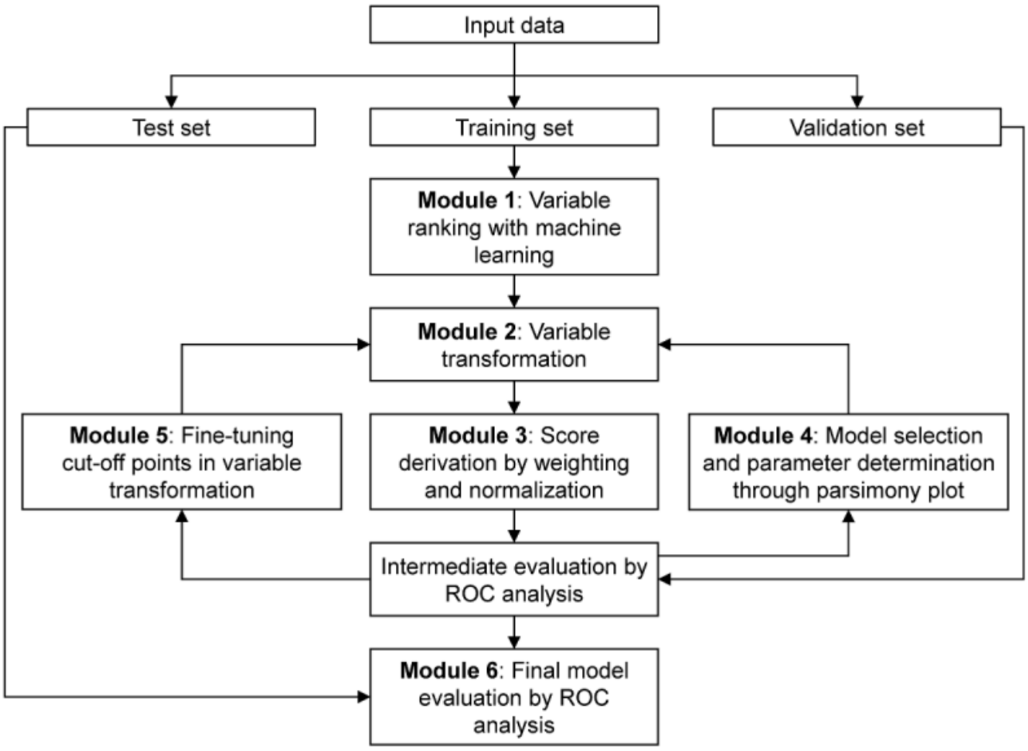

ROC: receiver operating characteristic.

© 2021 Xie F et al. *JAMA Network Open.*

eFigure 2. Parsimony Plot of the Number of Variables Versus AUC Values for (A) SERP-2d, (B) SERP-7d and (C) SERP-30d on the Validation Cohort

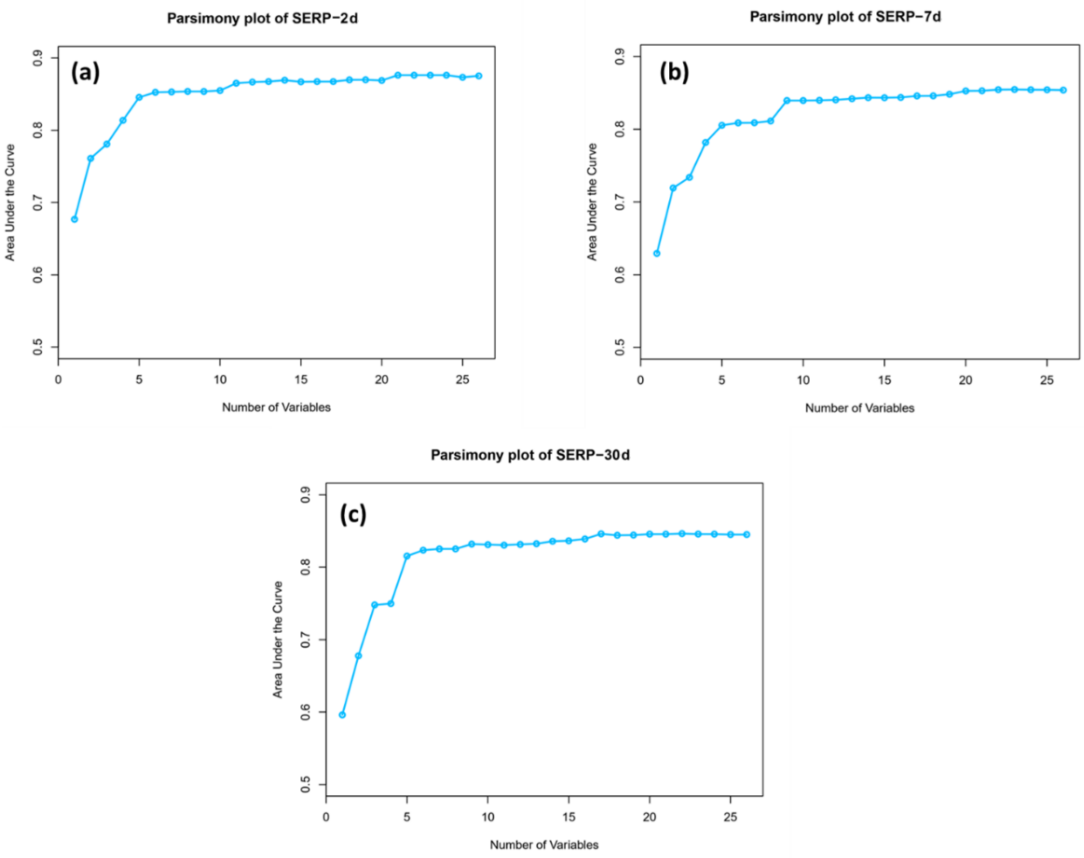

© 2021 Xie F et al. *JAMA Network Open.*

eFigure 3. Number of Cases Versus Score Value on the Testing Cohort for (A) SERP-2d and (B) SERP-30d

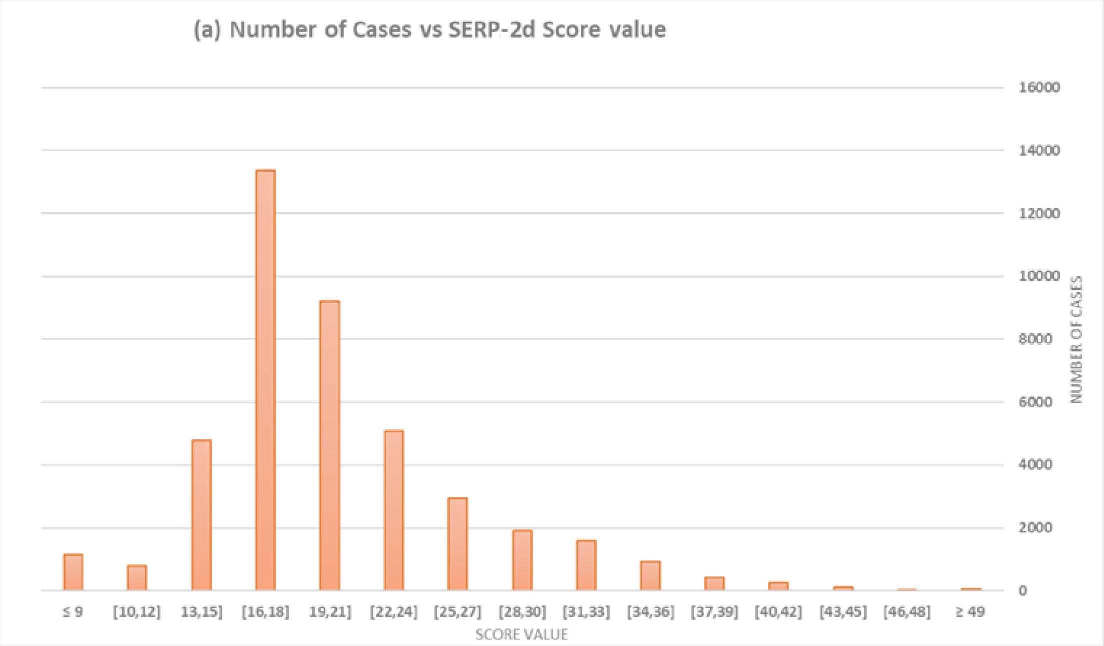

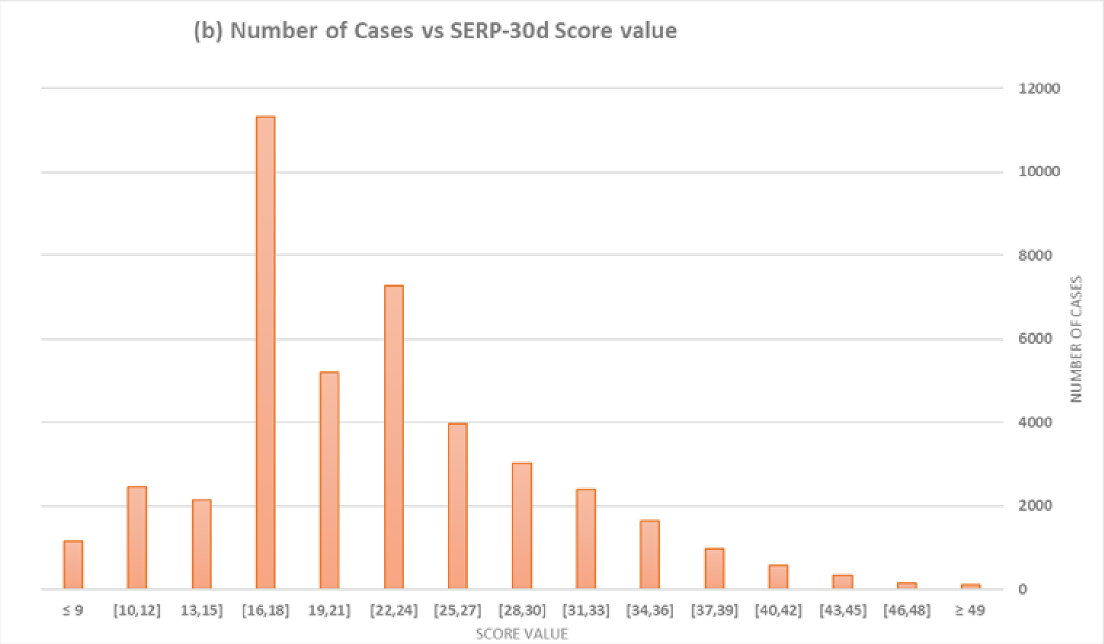

© 2021 Xie F et al. *JAMA Network Open.*

eFigure 4. Observed Mortality Rate Versus Score Value on the Testing Cohort for (A) SERP-2d and (B) SERP-30d

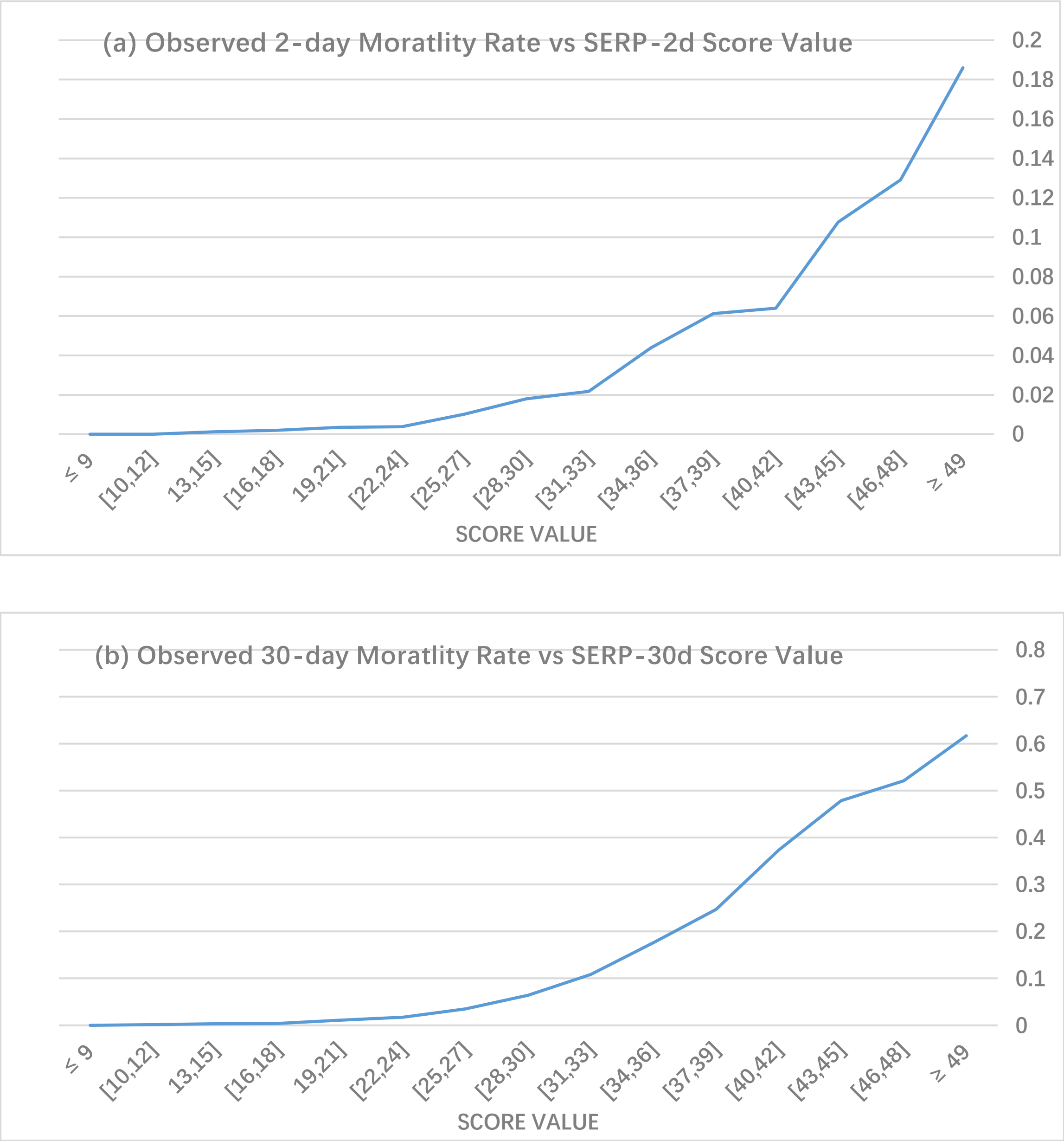

© 2021 Xie F et al. *JAMA Network Open.*

eMethods. Description of the AutoScore Method

Clinical risk-scoring models have been traditionally developed in 2 ways: through expert opinions/consensus and conventional cohort studies. However, both approaches are labor-intensive and are not easy to update over time. In order to develop a parsimonious model with easy access to validation in the context of EHRs, we used AutoScore, a generic method and novel framework to automate the development of a clinical scoring model for predefined outcomes. In this study, 3 SERP scores, namely SERP-2d, SERP-7d and SERP-30d, were developed using 3 primary outcomes of interest: 2-, 7-, and 30-day mortality, respectively.

AutoScore consists of six modules (eFigure 1): Module 1: variable ranking with machine learning, Module 2: variable transformation, Module 3: score derivation, Module 4: model selection, Module 5: domain knowledge-based score fine-tuning, and Module 6: performance evaluation. The details are described in the publication (http://dx.doi.org/10.2196/21798). Users (clinicians or scientists) could seamlessly generate parsimonious sparse-score risk models (i.e., risk scores), which can be easily implemented and validated in clinical practice. Also, it enables users to build transparent and straightforward clinical scores quickly. We hope to see its application in various medical case studies.

Following the flow of the AutoScore, the training cohorts initially went through the AutoScore algorithm, where the candidate variables were ranked in Module 1; continuous variables were discretized in Module 2; different categories were weighted in Module 3, and the candidate SERP scoring model was created.

Second, the number of variables was decided by the parsimony plot (i.e., model performance vs. complexity) (Module 4) based on the validation cohort. The parsimonious models would be selected while maximizing predictive accuracy.

Furthermore, the automatically generated cut-off values of each continuous variable can be fine-tuned by combining, rounding and adjusting according to the standard clinical norm. (Module 5)

Lastly, we confirmed the variables and fine-tuned cutoffs. Then Modules 2 and 3 would be re-run to generate the final SERP model. The performance final SERP model will be evaluated in Module 6.

© 2021 Xie F et al. *JAMA Network Open.*
